# Incontinence Quiz (IQ): Translation, Cross-Cultural Adaptation, and Psychometric Validation of the French Version

**DOI:** 10.3390/healthcare14101409

**Published:** 2026-05-20

**Authors:** Andrea Ribeiro, João Sousa, João Neves, Carla Macedo, José Lumini

**Affiliations:** 1 Centro Interdisciplinar em Ciências da Saúde (CICS), Instituto Superior de Saúde—ISAVE, 4720-155 Amares, Portugal; joaosousa@isave.pt (J.S.); joao.neves@docente.isave.pt (J.N.); carla.macedo@isave.pt (C.M.); jose.lumini@isave.pt (J.L.); 2 CIR, Escola Superior de Saúde, Instituto Politécnico do Porto, Rua Dr. António Bernardino de Almeida nº 400, 4200-072 Porto, Portugal; 3 Research Centre for Active Living and Wellbeing (Livewell), Instituto Politécnico de Bragança, 5300-253 Bragança, Portugal

**Keywords:** urinary incontinence, knowledge, questionnaire, psychometric validation, cross-cultural adaptation, women

## Abstract

**Highlights:**

**What are the main findings?**

- The French version of the Incontinence Quiz (IQ) showed acceptable internal consistency and good test–retest reliability.
- Exploratory Factor Analysis supported a multidimensional structure explaining 54.5% of the total variance.

**What are the implications of the main findings?**

- The French IQ can be used to assess knowledge about urinary incontinence in French-speaking women in educational and research settings.
- Availability of a validated French instrument facilitates international comparisons and the development of pelvic health educational interventions.

**Abstract:**

**Background/Objectives**: Urinary incontinence (UI) is common among women and is often underreported and undertreated, partly due to limited health literacy and persistent misconceptions regarding its causes and management. Instruments that reliably assess knowledge about UI are important for identifying educational needs and evaluating the impact of educational interventions. Although the Incontinence Quiz (IQ) has been validated in other languages, no psychometrically tested French version was previously available. This study aimed to translate, culturally adapt, and evaluate the measurement properties of the French version of the Incontinence Quiz (IQ-Fr) in adult women, following internationally recommended procedures for cross-cultural adaptation. **Methods**: A methodological validation study with a two-sample design was conducted. An extended sample (*n* = 289) was used to examine internal consistency and convergent validity, while a validation subsample (*n* = 40) was used to assess divergent validity and reproducibility. The translation process included forward translation, synthesis, back-translation, expert committee review, and pretesting. The internal consistency of the IQ-Fr was assessed using Cronbach’s Alpha. The convergent validity of the IQ-Fr was assessed by both Exploratory Factor Analysis (EFA) and Confirmatory Factor Analysis (CFA). The divergent validity of the IQ-Fr was evaluated by Pearson’s correlation IQ-Fr and Ditrovie quality-of-life scores. Finally, the reproducibility of the IQ-Fr was evaluated by Intraclass Correlation (ICC) between the IQ-Fr scores obtained at two different time points (T0 and T1) over a one-week interval. **Results**: The IQ-Fr showed acceptable internal consistency (Cronbach’s Alpha = 0.654) comparable to other translations/cultural adaptations made for the same instrument. The EFA and CFA suggest the same four-dimension structure (IQ-Fr) found in the original instrument (IQ), although the factorial model fit would benefit from the additional removal of item 6 from the questionnaire, as already suggested by the increase in the instrument’s Cronbach’s Alpha (from 0.646 to 0.659). The IQ-Fr also showed good divergent validity, as assessed by the absence of a statistically significant Pearson correlation between the scores of the IQ-Fr and the scores of a non-related construct—the Ditrovie scale (r_p_ = 0.097, *p*-value = 0.552). Lastly, the IQ-Fr showed good reproducibility, as demonstrated by the high ICC coefficient (ICC = 0.752) between the instrument’s overall scores at T0 and T1. **Conclusions**: The French version of the Incontinence Quiz (IQ-Fr) presents good indicators of internal consistency, convergent validity, divergent validity, and reproducibility for it to be used in research and educational contexts in French-speaking populations.

## 1. Introduction

Urinary incontinence (UI), defined as any involuntary loss of urine, is a highly prevalent condition among women across the lifespan and represents an important public health concern. Epidemiological studies estimate that between 25% and 45% of adult women experience UI, with prevalence increasing with age but remaining substantial in younger populations as well [1,2]. Beyond its physical symptoms, UI is associated with psychological distress, social withdrawal, reduced participation in daily and physical activities, and diminished quality of life, making it relevant not only to clinical care but also to broader health and social well-being [1,2,3].

Despite the availability of effective conservative treatments—such as pelvic floor muscle training and behavioral strategies recommended as first-line interventions—UI remains frequently underreported and undertreated [4]. Many women normalize symptoms as an inevitable consequence of ageing or childbirth, delay seeking care, or avoid discussing the problem with health professionals [5]. Stigma, embarrassment, misconceptions, and limited health literacy have been consistently identified as key barriers to disclosure and timely management [3,6,7]. These factors highlight the need for reliable tools to assess knowledge and misconceptions about UI, both for research and educational purposes.

Valid and culturally appropriate instruments are essential to identify educational needs, design targeted interventions, and evaluate their effectiveness [8]. The Incontinence Quiz (IQ), originally developed by Branch and colleagues, is a brief questionnaire designed to assess knowledge and beliefs related to UI, including its causes, treatments, and communication with healthcare professionals [5]. However, simple translation of instruments is insufficient; rigorous cross-cultural adaptation and psychometric validation are required to ensure conceptual equivalence, clarity, and measurement validity across languages and cultures [6,7,9,10]. A Portuguese version of the IQ has previously demonstrated acceptable reliability and reproducibility, supporting its use in educational and research contexts [11]. Nevertheless, no validated French version was available, despite French being widely used in clinical practice and research across Europe, Canada, and Africa. This represents a relevant gap for the standardized assessment of UI-related knowledge in French-speaking populations.

Therefore, the aim of this study was to translate and culturally adapt the Incontinence Quiz into French and to evaluate its psychometric properties in adult women, including internal consistency, internal structure, construct validity, divergent validity, and test–retest reliability. Overall, the findings indicate that the French version of the IQ presents acceptable reliability and supportive evidence of validity for use in research and educational settings.

## 2. Materials and Methods

### 2.1. Study Design

This study was a methodological cross-cultural adaptation and psychometric validation of a health-related knowledge questionnaire. The study followed internationally accepted guidelines for translation and validation of patient-reported outcome measures, including structured translation procedures and evaluation of measurement properties (reliability and validity) [9,10,12]. This study was reported in accordance with the STROBE (Strengthening the Reporting of Observational Studies in Epidemiology) guidelines for cross-sectional studies and the COSMIN recommendations for studies on measurement properties of patient-reported outcome measures [13,14]. A completed STROBE checklist is provided as Supplementary Materials.

### 2.2. Participants

In agreement with the demographic statistics published by the National Institute of Statistics and Economic Studies (INSEE), France has currently around 70 million inhabitants.

The “Sample Size Calculator” tool on the “Calculator.net” website was used to assess the minimum sample size required for the present study. Assuming a 5% error margin (95% confidence level) and a 25% proportion of French-fluent adult women in France, a minimum sample size of 289 elements was suggested to meet the desired statistical constraints.

An extended sample comprising 289 French adult women was used for the evaluation of content validity (critical analysis by a panel of experts), internal consistency (Cronbach’s Alpha), and convergent validity (structural analysis by Exploratory and Confirmatory Factor Analysis).

A validation subsample of 40 French adult women extracted from the original extended sample was used for evaluating divergent (discriminant) validity (comparison with a non-related construct: Ditrovie scale) and reproducibility (test–retest reliability).

Inclusion criteria considered in the present research included age ≥ 18 years, fluency in French, ability to provide informed consent, and complete IQ responses.

### 2.3. Data Collection Instruments

The data collection form, self-administered, consisted of three sections:

▪ Sociodemographic and clinical characterization of the sample.
▪ French version of the Incontinence Quiz (IQ-Fr): a 14-item questionnaire originally developed to assess knowledge and beliefs about urinary incontinence (UI) [5]. Items are presented as statements to which participants respond “agree”, “disagree”, or “don’t know”. One point is awarded for each correct answer, yielding a total score ranging from 0 to 14, with higher scores indicating greater knowledge. Conceptually, items cover four domains [5]: (1) ageing and UI, (2) causes of UI, (3) communication with health professionals, and (4) treatments and consequences of UI. No modifications in the structure and scoring system of the original instrument (IQ) were made in the translated and cross-culturally adapted version of the IQ (IQ-Fr).
▪ Ditrovie questionnaire (only to be applied to the validation subsample for evaluating divergent validity and reproducibility): a self-administered, 10-item French tool validated to measure how urinary urge incontinence affects a patient’s quality of life. It assesses five domains—activity, self-image, emotional impact, sleep, and general well-being—using a negative scale from 1 (best) to 5 (worst) to evaluate treatment outcomes and clinical symptoms.

### 2.4. Procedure—Translation and Cross-Cultural Adaptation

The translation and cultural adaptation process of the IQ to the IQ-Fr followed standardized international recommendations [9,10]. The procedure included the following:

1. Forward translation: Two independent bilingual translators produced separate French translations of the original IQ.
2. Synthesis: The two translations were compared and merged into a single reconciled version.
3. Back-translation: An independent translator, blinded to the original questionnaire, translated the synthesized French version back into English.
4. Expert committee review: A multidisciplinary panel (including physiotherapists, a methodologist, and a language specialist) reviewed all versions to ensure semantic, idiomatic, experiential, and conceptual equivalence.
5. Pretesting: The pre-final version was tested with a small group of French-speaking women to assess clarity, comprehension, and cultural relevance. Minor wording adjustments were made accordingly.

### 2.5. Procedure—Ethical Aspects

This study was conducted in accordance with the Declaration of Helsinki. Ethical approval was granted by the ISAVE Ethics Committee (Amares, Portugal) in 2024 (research project approval no. 2024/03-01). All participants provided informed consent prior to their participation the study.

The datasets generated and analyzed during the current study are not publicly available due to institutional data protection policies, but they are available from the corresponding author upon reasonable request.

### 2.6. Procedure—Statistical Analysis

IBM SPSS Statistics v30 and IBM SPSS Amos v25 were used for the statistical data analysis of the collected data. Descriptive statistics for each studied variable involved the determination of a measure of central tendency (mode for nominal variables, median for ordinal variables, and mean for numeric variables), a measure of dispersion (frequencies for nominal variables, interquartile range for ordinal variables, and standard deviation for numeric variables), and a form of representation (bar chart for nominal and ordinal variables, histogram for numeric variables).

The internal consistency of the IQ-Fr was assessed by Cronbach’s Alpha (α), with a minimum acceptable value of around 70% for the instrument to be considered for exploratory purposes.

The convergent validity of the IQ-Fr was assessed by both Exploratory Factor Analysis (EFA) and Confirmatory Factor Analysis (CFA). EFA of the IQ-Fr was conducted in three sequential steps: (i) determination of the number of factors to extract from the study variables; (ii) rotation of the extracted factors; and (iii) interpretation of the resulting factor matrix. The adequacy of the factor analysis was assessed by the following criteria: (i) Bartlett’s Test of Sphericity (*p*-value < 0.05 indicates statistically significant correlations among variables); (ii) Kaiser–Meyer–Olkin (KMO) index (value ≥ 0.50 indicates sufficient shared variance among variables); (iii) determinant of the correlation matrix (value above 1 × 10^−5^ indicates the absence of severe multicollinearity); and (iv) adequate sample size (ideally at least 40 observations per variable). To determine the appropriate number of factors to retain, Principal Component Analysis (PCA) was used as the extraction method, alongside the following evaluation criteria: eigenvalues ≥ 1, inspection of the scree plot (to identify the point of inflection), and retention of factors explaining at least 60% of the total variance. Factor rotation was performed using the orthogonal Varimax method with Kaiser normalization. In the final factor matrix, loadings ≥0.40 were considered meaningful. Variables exhibiting cross-loadings or failing to present loadings ≥ 0.40 on any factor were excluded from the model analysis.

CFA of the IQ-Fr was conducted using the four-factor structure of the original IQ instrument: domain 1 (“Relationship between aging and UI”) comprising items 1 and 2; domain 2 (“Causes of UI”) comprising items 3, 8, 10, and 12; domain 3 (“Physician–patient discussions about UI”) comprising items 7 and 9; and domain 4 (“Treatments and effects of UI”) comprising items 4, 5, 6, 11, 13, and 14. The overall model fit was evaluated using multiple goodness-of-fit indices, including the Chi-square test (*p*-value > 0.05 indicates good fit), Comparative Fit Index (CFI; acceptable ≥ 0.90, excellent ≥ 0.95), Tucker–Lewis Index (TLI ≥ 0.90), Root Mean Square Error of Approximation (RMSEA; acceptable < 0.08, good < 0.06), and Standardized Root Mean Square Residual (SRMR < 0.08). Model refinement was performed and guided by the modification indices provided by the statistical analysis software.

The divergent (discriminant) validity of the IQ-Fr was assessed by Pearson’s correlation coefficient between the IQ-Fr and Ditrovie scores, with statistical significance set at the 5% level (*p* < 0.05). The absence of a statistically significant linear correlation between the two instruments was considered to be evidence in support of the divergent validity of the IQ-Fr.

Finally, the reproducibility (test–retest reliability) of the IQ-Fr was evaluated by Intraclass Correlation Coefficient (ICC) based on the IQ-Fr scores obtained at two different time points (T0 and T1). The setup for the ICC analysis included a two-way mixed model, absolute agreement type, with single measures. ICC values above 0.75 for the IQ-Fr scores were considered an indicator of good reproducibility (test–retest reliability) in the instrument.

### 2.7. Use of Generative Artificial Intelligence (GenAI)

No generative artificial intelligence tools were used for the study design, data collection, statistical analysis, or interpretation of the results.

## 3. Results

### 3.1. Clinical and Sociodemographic Overview of the Participants

Table 1, below, summarizes the sociodemographic and clinic characterization of both the extended sample comprising 289 French adult women used in the evaluation of the IQ-Fr’s content validity, internal consistency, and convergent validity, and the subsample of 40 French adult women used in the evaluation of the IQ-Fr’s divergent (discriminant) validity and reproducibility (test–retest reliability).

No significant statistical differences have been detected between the extended sample (n = 289) and the validation subsample (n = 40) concerning the sociodemographic and clinic characterization variables studied, which demonstrates the coherence of the statistical analysis procedure adopted.

Regarding the IQ-Fr results, the sample participants show relatively high levels of literacy in the domain “Treatments and effects of UI” (62.92% of maximum score), intermediate levels of literacy in the domain “Causes of UI” (47.49% of maximum score), and low levels of literacy in the domains “Physician–patient discussions about UI” and “Relationship between aging and UI” (37.89% and 30.45%, respectively, of maximum score). The overall score of adult French women in the IQ-Fr instrument shows an intermediate level of literacy regarding UI (50.30% of maximum score).

### 3.2. Internal Consistency of IQ-Fr (Cronbach’s Alpha)

The 14-item IQ-Fr instrument shows an overall Cronbach’s Alpha (α) of 0.646, indicating an acceptable internal consistency given the multidimensional nature of the construct and the reduced number of items (two characteristics that tend to underestimate the true internal consistency). The following Table 2 presents the corrected item–total correlation and Cronbach’s Alpha (α) if item deleted.

Only the elimination of item 6 in the IQ-Fr should be considered due to the low corrected item–total correlation (0.079) and the increase in Cronbach’s Alpha of the IQ-Fr (from 0.646 to 0.659) upon its removal.

### 3.3. Convergent Validity of IQ-Fr (EFA and CFA)

The values of the KMO Index (0.700), Bartlett’s Test of Sphericity (χ^2^ = 426.221, *p*-value < 0.001), and the determinant of the correlation matrix (0.221) jointly show that the dataset is appropriate for factor analysis (EFA and CFA).

For EFA, although the criteria of eigenvalues ≥ 1 and total variance ≥ 60% suggest a five-dimension structure of the IQ-Fr, the scree plot point of inflection (the most determinant parameter in deciding the number of factors to extract in EFA) points to a four-dimension structure, in agreement with the original IQ instrument. A four-factor structure of the IQ-Fr was therefore considered for the instrument. After Varimax factorial rotation with Kaiser normalization, the rotated component matrix generated by EFA was as follows in Table 3.

The analysis of the IQ-Fr’s rotated component matrix generated by EFA shows the same allocation of items to the four factors extracted, as viewed in the original IQ instrument, with the exception of item 6. These results suggest, once again, the possibility of eliminating item 6 from the IQ-Fr in order to increase the psychometric properties of the instrument.

**Figure 1:**
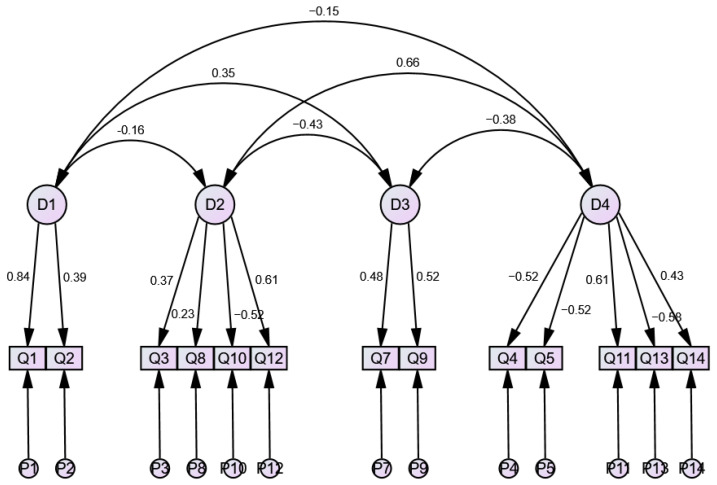
Final suggested structure of IQ-Fr (4 domains, 13 items after elimination of item 6), as generated by CFA.

On the basis of item 6’s removal from the IQ-Fr, CFA for the instrument was performed using the same four-dimension structure of the original IQ instrument, but only considering the remaining 13 items. The final suggested structure for the IQ-Fr, as indicated by Cronbach’s Alpha (α) and CFA, is as follows:

The final suggested structure of the IQ-Fr presented in Figure 1 shows good overall model fit, as evaluated by the following values of CFA’s quality of adjustment parameters: Chi-square test (χ^2^= 65.561, *p*-value = 0.260), Comparative Fit Index (CFI = 0.978), Tucker–Lewis Index (TLI = 0.971), Root Mean Square Error of Approximation (RMSEA = 0.021), and Standardized Root Mean Square Residual (SRMR = 0.010). No model refinement was needed in order to obtain the above presented values.

### 3.4. Divergent Validity of IQ-Fr (Pearson’s Correlation Coefficient)

The divergent (discriminant) validity of the IQ-Fr was evaluated by direct comparison of the IQ-Fr with a non-related construct (Ditrovie scale). The determination of Pearson’s correlation coefficient between the IQ-Fr’s overall score and the Ditrovie scale’s quality-of-life mean score showed the absence of any statistically significant correlation between the instruments (r_p_ = 0.097, *p*-value = 0.552). This supports divergent validity, indicating that knowledge about urinary incontinence (IQ-Fr) is distinct from symptom-related quality of life.

### 3.5. Reproducibility of IQ-Fr (Intraclass Correlation Coefficient)

The reproducibility (test–retest reliability) of the IQ-Fr was evaluated by Intraclass Correlation Coefficient (ICC) based on the different IQ-Fr scores obtained at the two different time points (T0 and T1) tested.

With the exception of the IQ-Fr score for the dimension “Treatments and effects of UI” (ICC = 0.653, which is, however, considered acceptable in the literature, as it is higher than 0.50), all the remaining IF-Fr scores present ICC values above the established threshold of 0.75. Altogether, the ICC values presented in Table 4 show that this translated and culturally adapted version of the IQ instrument for the French adult women population (IQ-Fr) presents good reproducibility (test–retest reliability).

## 4. Discussion

This study presents the translated and culturally adapted version of the original 14-item Incontinence Quiz (IQ) to the French-speaking adult women population residing in France (IQ-Fr), while evaluating its psychometric properties (internal consistency, convergent validity, divergent validity, and reproducibility) [5,11,15,16].

The 14-item IQ-Fr shows an acceptable internal consistency (Cronbach’s Alpha = 0.646) that, although slightly below the conventional threshold of 0.70 found in the literature, remains within an acceptable range for this type of short, multidimensional literacy evaluation instrument, where internal consistency tends to be underestimated [8]. The elimination of item 6 from the IQ-Fr is suggested by the low corrected item–total correlation (0.079) of item 6 and by the increase in the instrument’s Cronbach’s Alpha after item 6’s removal, from 0.646 to 0.659. Similar values of Cronbach’s Alpha have been reported in previous validations of the IQ, including the Portuguese version of the IQ [11], suggesting that this level of internal consistency reflects the conceptual breadth of the instrument rather than the presence of methodological weakness [8].

The 14-item IQ-Fr also presents a good convergent validity, as evaluated by EFA, denoting the same four-dimension structure found in the original IQ instrument with satisfactory model fit to the dataset. Interestingly, the rotated component matrix generated by EFA also suggests the elimination of item 6 from the IQ-Fr, as it is the sole item wrongly allocated to a factor: in the IQ-Fr, item 6 is allocated to the domain “Causes of UI” when, in the original IQ instrument, it belongs to the domain “Treatments and effects of UI”. This possibility was further explored in the CFA by evaluating the convergent validity of a 13-item IQ-Fr (without item 6), which showed an excellent overall model fit: Chi-square test (χ^2^= 65.561, *p*-value = 0.260), Comparative Fit Index (CFI = 0.978), Tucker–Lewis Index (TLI = 0.971), Root Mean Square Error of Approximation (RMSEA = 0.021), and Standardized Root Mean Square Residual (SRMR = 0.010).

Additionally, the IQ-Fr shows good divergent validity, as confirmed by the absence of a statistically significant linear correlation (r_p_ = 0.097, *p*-value = 0.552) between the IQ-Fr overall score and the overall score of a non-related construct (Ditrovie scale, quality-of-life measurement). As expected, knowledge about UI is conceptually distinct from symptom severity or impact on daily life [3]. This result confirms that the IQ-Fr measures knowledge rather than lived experience or clinical burden, which is essential for its intended use in educational and research settings.

Finally, the IQ-Fr also denotes high reproducibility (test–retest reliability) of measurement in the two time points tested (T0 and T1) over a one-week interval. Intraclass Correlation Coefficients (ICCs) above the established threshold of 0.75 have been obtained for all the IQ-Fr’s scores (domains and global), with the exception of the IQ-Fr score for the domain “Treatments and effects of UI” (ICC = 0.653, which is, however, considered acceptable in the literature, as it is higher than 0.50) [17,18].

## 5. Conclusions

This translated and culturally adapted version of the original IQ to the French-speaking adult women population (IQ-Fr) presents good psychometric properties (internal consistency, convergent validity, divergent validity, and reproducibility) to be considered for exploratory purposes.

From a broader perspective, the availability of this validated French version of the IQ is particularly relevant given the widespread use of French in healthcare and research across Europe, Canada, and Africa. The IQ-Fr can facilitate cross-cultural comparisons, support multinational studies on pelvic health literacy, and inform the design and evaluation of educational interventions aimed at improving awareness, help-seeking behavior, and uptake of conservative treatments for UI [4,7].

Two important limitations should be acknowledged. First, convenience sampling may limit generalizability to the broader population of French-speaking women. Second, the evaluation of divergent validity and reproducibility of the IQ-Fr was performed in smaller subsamples of participants extracted from the extended sample of 289 participants, which could limit the statistical significance of the results [10,12].

In conclusion, the present study provides evidence that the French version of the Incontinence Quiz is a reliable and valid instrument for assessing knowledge about urinary incontinence in adult women. Future research should further explore its utility in clinical education, public health initiatives, and intervention studies aimed at improving pelvic health literacy.

## Figures and Tables

**Table 1:** Sociodemographic and clinic characterization of the sample.

Variable	Extended Sample	Validation Subsample	Difference Testing (*p*-Value)
N	289	40	*p* > 0.05
Age (Mean ± SD)	35.58 ± 13.62	36.15 ± 14.60	*p* > 0.05
IQ-Fr—Q1 (Count (%))	Correct: 106 (36.7%) Incorrect: 183 (63.3%)	Correct: 13 (32.5%) Incorrect: 27 (67.5%)	*p* > 0.05
IQ-Fr—Q2 (Count (%))	Correct: 70 (24.2%) Incorrect: 219 (75.8%)	Correct: 15 (37.5%) Incorrect: 25 (62.5%)	*p* > 0.05
IQ-Fr—Q3 (Count (%))	Correct: 90 (31.1%) Incorrect: 199 (68.9%)	Correct: 11 (27.5%) Incorrect: 29 (72.5%)	*p* > 0.05
IQ-Fr—Q4 (Count (%))	Correct: 173 (59.9%) Incorrect: 116 (41.1%)	Correct: 22 (55.0%) Incorrect: 18 (45.0%)	*p* > 0.05
IQ-Fr—Q5 (Count (%))	Correct: 188 (65.1%) Incorrect: 101 (34.9%)	Correct: 24 (60.0%) Incorrect: 16 (40.0%)	*p* > 0.05
IQ-Fr—Q6 (Count (%))	Correct: 111 (38.4%) Incorrect: 178 (61.6%)	Correct: 18 (55.0%) Incorrect: 22 (45.0%)	*p* > 0.05
IQ-Fr—Q7 (Count (%))	Correct: 39 (13.5%) Incorrect: 250 (86.5%)	Correct: 4 (10.0%) Incorrect: 36 (90.0%)	*p* > 0.05
IQ-Fr—Q8 (Count (%))	Correct: 201 (69.6%) Incorrect: 88 (30.4%)	Correct: 27 (67.5%) Incorrect: 13 (32.5%)	*p* > 0.05
IQ-Fr—Q9 (Count (%))	Correct: 180 (62.3%) Incorrect: 109 (37.7%)	Correct: 26 (65.0%) Incorrect: 14 (35.0%)	*p* > 0.05
IQ-Fr—Q10 (Count (%))	Correct: 132 (45.7%) Incorrect: 157 (54.3%)	Correct: 18 (45.0%) Incorrect: 22 (55.0%)	*p* > 0.05
IQ-Fr—Q11 (Count (%))	Correct: 191 (66.1%) Incorrect: 98 (33.9%)	Correct: 25 (62.5%) Incorrect: 15 (37.5%)	*p* > 0.05
IQ-Fr—Q12 (Count (%))	Correct: 126 (43.6%) Incorrect: 163 (56.4%)	Correct: 14 (35.0%) Incorrect: 26 (65.0%)	*p* > 0.05
IQ-Fr—Q13 (Count (%))	Correct: 160 (55.4%) Incorrect: 129 (44.6%)	Correct: 21 (52.5%) Incorrect: 19 (47.5%)	*p* > 0.05
IQ-Fr—Q14 (Count (%))	Correct: 268 (92.7%) Incorrect: 21 (7.3%)	Correct: 40 (100.0%) Incorrect: 0 (0.0%)	*p* > 0.05
IQ-Fr—% Maximum Score in “Relationship between aging and UI” Domain (Mean ± SD)	30.45 ± 36.90	35.00 ± 37.89	*p* > 0.05
IQ-Fr—% Maximum Score in “Causes of UI” Domain (Mean ± SD)	47.49 ± 29.61	46.25 ± 28.05	*p* > 0.05
IQ-Fr—% Maximum Score in “Physician–patient discussions about UI” Domain (Mean ± SD)	37.89 ± 32.95	37.50 ± 31.52	*p* > 0.05
IQ-Fr—% Maximum Score in “Treatments and effects of UI” Domain (Mean ± SD)	62.92 ± 25.65	62.50 ± 26.35	*p* > 0.05
IQ-Fr–IQ-Fr—% Maximum Overall Score (Mean ± SD)	50.30 ± 19.36	50.36 ± 19.41	*p* > 0.05
Ditrovie—Mean Score (Mean ± SD)	Not applicable	1.25 ± 0.58	Not applicable

**Table 2:** IQ-Fr corrected item–total correlation and Cronbach’s Alpha (α) if item deleted.

Variable	Corrected Item–Total Correlation	Cronbach’s Alpha (α) if Item Deleted
IQ-Fr—Q1	0.185	0.642
IQ-Fr—Q2	0.190	0.640
IQ-Fr—Q3	0.133	0.650
IQ-Fr—Q4	0.346	0.616
IQ-Fr—Q5	0.316	0.621
IQ-Fr—Q6	0.079	0.659
IQ-Fr—Q7	0.204	0.638
IQ-Fr—Q8	0.188	0.641
IQ-Fr—Q9	0.240	0.634
IQ-Fr—Q10	0.396	0.607
IQ-Fr—Q11	0.392	0.608
IQ-Fr—Q12	0.416	0.603
IQ-Fr—Q13	0.440	0.599
IQ-Fr—Q14	0.282	0.632

**Table 3:** IQ-Fr item loadings in the rotated component matrix generated by EFA for a 4-factor structure.

Item	Item Loadings			
	Factor 1	Factor 2	Factor 3	Factor 4
IQ-Fr—Q1	0.738	−0.134	0.242	0.067
IQ-Fr—Q2	0.819	0.126	−0.076	0.028
IQ-Fr—Q3	−0.148	0.628	0.255	−0.104
IQ-Fr—Q4	0.129	−0.050	0.046	0.659
IQ-Fr—Q5	−0.121	0.019	0.094	0.657
IQ-Fr—Q6	−0.056	0.554	−0.181	−0.005
IQ-Fr—Q7	0.090	−0.165	0.753	0.128
IQ-Fr—Q8	0.083	0.414	−0.181	0.192
IQ-Fr—Q9	0.033	0.185	0.666	0.060
IQ-Fr—Q10	0.133	0.563	0.345	0.179
IQ-Fr—Q11	0.002	0.069	0.019	0.710
IQ-Fr—Q12	0.135	0.466	0.159	0.375
IQ-Fr—Q13	0.274	0.139	0.045	0.606
IQ-Fr—Q14	−0.015	0.124	0.036	0.493

**Table 4:** Intraclass Correlation Coefficient (ICC) of IQ-Fr scores at T0 and T1.

IQ-Fr Scores	T0	T1	ICC
IQ-Fr—% Maximum Score in “Relationship between aging and UI” Domain (Mean ± SD)	25.00 ± 35.36	35.00 ± 41.16	0.833
IQ-Fr—% Maximum Score in “Causes of UI” Domain (Mean ± SD)	52.50 ± 29.93	52.50 ± 36.23	0.827
IQ-Fr—% Maximum Score in “Physician–patient discussions about UI” Domain (Mean ± SD)	35.00 ± 33.75	40.0 ± 31.62	0.883
IQ-Fr—% Maximum Score in “Treatments and effects of UI” Domain (Mean ± SD)	66.67 ± 22.22	71.67 ± 23.63	0.653
IQ-Fr–IQ-Fr—% Maximum Overall Score (Mean ± SD)	52.14 ± 20.77	56.43 ± 16.31	0.752

## Data Availability

The datasets generated during this study are not publicly available due to institutional data protection and participant privacy requirements but are available from the corresponding author upon reasonable request.

## Supplementary Materials

The following supporting information can be downloaded at https://www.mdpi.com/article/10.3390/healthcare14101409/s1, STROBE Checklist.
