# Supplementary material for: Incontinence Quiz (IQ): Translation, Cross-Cultural Adaptation, and Psychometric Validation of the French Version
Source: Healthcare (Basel). 2026 May 20;14(10):1409. doi: 10.3390/healthcare14101409 (PMC13207247; doi:10.3390/healthcare14101409)
Supplement: Supplementary file 1 [file healthcare-14-01409-s001.zip › healthcare-4233310-supplementary.pdf]

Supplementary Materials: The STROBE checklist for cross-sectional studies is provided as supplementary material.

STROBE Checklist (Cross-Sectional Studies)

Manuscript: *Incontinence Quiz (IQ): Translation, Cross-Cultural Adaptation, and Psychometric Validation of the French Version*

Section	Item	STROBE recommendation	Where addressed in manuscript
Title and Abstract	1	Indicate the study design with a commonly used term in the title or abstract	Title and Abstract
Abstract	2	Provide an informative and balanced summary of what was done and what was found	Abstract
Introduction	3	Explain the scientific background and rationale for the investigation	Introduction
Introduction	4	State specific objectives or hypotheses	End of Introduction
Methods	5	Present key elements of study design early in the paper	Section 2.1 Study Design
Methods	6	Describe the setting, locations, and relevant dates	Section 2.4 Participants
Methods	7	Give eligibility criteria and sources/methods of participant selection	Section 2.4 Participants
Methods	8	Clearly define outcomes, exposures, predictors, and confounders	Sections 2.2 Instrument and 2.5 Data Collection
Methods	9	Describe data sources and measurement methods	Section 2.5 Data Collection
Methods	10	Describe efforts to address potential sources of bias	Section 2.1 Study Design and 2.6 Statistical Analysis
Methods	11	Explain how the study size was determined	Section 2.4 Participants
Methods	12	Describe all statistical methods used	Section 2.6 Statistical Analysis
Results	13	Report numbers of individuals at each stage of the study	Results—Participants description
Results	14	Provide characteristics of study participants	Results—Descriptive statistics
Results	15	Report numbers of outcome events or summary measures	Results—Psychometric analyses
Results	16	Provide main results including estimates and precision	Results—Validity and reliability analyses
Results	17	Report other analyses performed	Results—Factor analysis
Discussion	18	Summarise key results with reference to study objectives	Discussion—first paragraph
Discussion	19	Discuss limitations of the study	Discussion—limitations paragraph
Discussion	20	Provide overall interpretation of results	Discussion
Discussion	21	Discuss generalisability of findings	Discussion—last paragraphs
Other information	22	Give source of funding and ethical approval	Ethics approval section
